# Comparative Toxicity Assessment of Kratom Decoction, Mitragynine and Speciociliatine Versus Morphine on Zebrafish (*Danio rerio*) Embryos

**DOI:** 10.3389/fphar.2021.714918

**Published:** 2021-08-20

**Authors:** Thenmoly Damodaran, Nelson Jeng-Yeou Chear, Vikneswaran Murugaiyah, Mohd Nizam Mordi, Surash Ramanathan

**Affiliations:** ^1^ Centre for Drug Research, Universiti Sains Malaysia, George Town, Malaysia; ^2^ Discipline of Pharmacology, School of Pharmaceutical Sciences, Universiti Sains Malaysia, George Town, Malaysia

**Keywords:** kratom, mitragynine, speciociliatine, morphine, zebrafish, embryotoxicity

## Abstract

**Background:** Kratom (*Mitragyna speciosa* Korth), a popular opioid-like plant holds its therapeutic potential in pain management and opioid dependence. However, there are growing concerns about the safety or potential toxicity risk of kratom after prolonged use.

**Aim of the study:** The study aimed to assess the possible toxic effects of kratom decoction and its major alkaloids, mitragynine, and speciociliatine in comparison to morphine in an embryonic zebrafish model.

**Methods:** The zebrafish embryos were exposed to kratom decoction (1,000–62.5 μg/ml), mitragynine, speciociliatine, and morphine (100–3.125 μg/ml) for 96 h post-fertilization (hpf). The toxicity parameters, namely mortality, hatching rate, heart rate, and morphological malformations were examined at 24, 48, 72, and 96 hpf, respectively.

**Results:** Kratom decoction at a concentration range of ≥500 μg/ml caused 100% mortality of zebrafish embryos and decreased the hatching rate in a concentration-dependent manner. Meanwhile, mitragynine and speciociliatine exposure resulted in 100% mortality of zebrafish embryos at 100 μg/ml. Both alkaloids caused significant alterations in the morphological development of zebrafish embryos including hatching inhibition and spinal curvature (scoliosis) at the highest concentration. While exposure to morphine induced significant morphological malformations such as pericardial oedema, spinal curvature (lordosis), and yolk edema in zebrafish embryos.

**Conclusion:** Our findings provide evidence for embryonic developmental toxicity of kratom decoction and its alkaloids both mitragynine and speciociliatine at the highest concentration, hence suggesting that kratom consumption may have potential teratogenicity risk during pregnancy and thereby warrants further investigations.

## Introduction

*Mitragyna speciosa* Korth*.* (*Rubiaceae*), is known as Ketum or Biak-biak in Malaysia and Kratom in Thailand. This plant holds various therapeutic potentials especially in pain management and opioid dependence (Adkins et al., 2011; Cinosi et al., 2015). People in the countryside often consumed kratom in the form of a decoction, where mature fresh leaves are harvested and brewed for several hours. . Kratom leaves can also be chewed, smoked, and ingested as a solution or taken with tea/coffee (Assanangkronchai et al., 2007; Singh et al., 2016). Given its curative properties, kratom leaves are traditionally used to treat pain, diabetes, and diarrhea (Hassan et al., 2013; Singh et al., 2016). In fact, it is also used to enhance mood and ameliorate opioid withdrawal among illicit opioid users (Vicknasingam et al., 2010; Saraf et al., 2019).

Kratom leaves contains more than 40 indole alkaloids and is reported to produce unique pharmacological effects through its complex synergistic or antagonistic interactions (Obeng et al., 2020; Chear et al., 2021). Among these, mitragynine is the major alkaloid found in kratom leaves. Other active alkaloids present in kratom leaves are 7-hydroxymitragynine, speciogynine, speciociliatine, and paynantheine (Sharma et al., 2019; Chear et al., 2021). Mitragynine is known to reduce pain in preclinical evaluations (Matsumoto et al., 1996; Carpenter et al., 2016). In a recent randomized, double-blind placebo clinical trial, kratom decoction is shown to have the potential to suppress pain, however further clinical studies are needed to support its utility (Vicknasingam et al., 2020). Lately, we reported that speciociliatine showed a better binding affinity (*Ki* = 54.5 nM) towards the human mu-opioid receptor compared to its diastereoisomer—mitragynine (*Ki* = 161 nM). This alkaloid constitutes 9% of the total alkaloid present in kratom leaves. Further to this, speciociliatine demonstrated a better antinociceptive effect in rats when compared to mitragynine (Obeng et al., 2020). Taken together, these findings indicate that mitragynine along with speciociliatine could be a potential drug candidate for opioid substitution therapy and pain treatment.

The Zebrafish (*Danio rerio*), a small aquatic vertebrate, is a valid translational model in the field of neuroscience research, toxicology, or translational medicine (Gut et al., 2017; Vaz et al., 2018; Cassar et al., 2019). Importantly, zebrafish shared about 70% of human genes, and about 84% of genes known to human diseases are also present in zebrafish (Howe et al., 2013). Additionally, zebrafish also shared physiological and anatomical similarities in cardiovascular, nervous, and digestive systems with mammals (Hsu et al., 2007). Besides that, zebrafish have become a preferred animal model for extensive drug discovery research due to their small size, high fecundity, optical transparency, and fast development (Strähle et al., 2012; Vaz et al., 2018). More importantly, the zebrafish embryo has served as a promising model for screening toxicants that affect early embryonic development because of its comparable cell structure (i.e. embryonic yolk sac), and development pathway with humans (Link and Megason, 2008; Sant and Timme-Laragy, 2018). In fact, the external development of zebrafish embryos offers a great advantage to overcome the limitation of observing minute changes caused by toxicants during the early embryonic development due to the involvement of the maternal system in humans (He et al., 2012). The outcomes of various toxicity studies indicate that zebrafish embryos is a valuable animal model to anticipate the acute toxicity and teratogenicity effects of natural products/drugs in mammals especially humans (Strähle et al., 2012; Chakraborty et al., 2016; Blahova et al., 2020; Mektrirat et al., 2020).

Previously we tested kratom decoction for its efficacy in mitigating pain in regular kratom users. Despite it being consumed widely as a decoction, the preclinical toxicity data on kratom decoction and its active alkaloids remains limited and urgently warrants further investigations to support propspective human trial studies. Given this, the present study aimed to investigate the toxic effects of kratom decoction and its two active alkaloids mitragynine and speciociliatine on zebrafish embryos. Since kratom is shown to have morphine-like effects, we also assess the toxic effects of morphine concurrently with mitragynine and speciociliatine for comparison purposes.

## Methods

### Zebrafish Husbandry and Breeding

Zebrafish (*Danio rerio*), wild-type AB strain were used in this study. The zebrafish were maintained in the automated housing system (Tecniplast, Italy) that automatically regulates the pH (7.5 ± 0.5), temperature (28°C ±0.5), salinity, and water flow with 14 h light:10 h dark cycle (light onset: 8 am; light offset: 10 pm). They were fed daily with tetraMin^®^ tropical flakes and live brine shrimp twice per day. Embryos were obtained from spawning sexually matured male and female adult zebrafish at a ratio of 2:2 through natural mating. According to European legislation (EU Directive, 2010/63/EU), no animal ethics permission was requested for zebrafish larvae below 120 h post-fertilization (hpf) (Strähle et al., 2012).

### Plant Materials

Approximately 4 kg of fresh kratom (*Mitragyna speciosa* Korth.) leaves were collected from a local farm located at Permatang Rawa, Penang, Malaysia. The plant was authenticated by a botanist, Dr. Rosazlina binti Rusly from the School of Biological Sciences, Universiti Sains Malaysia. A voucher specimen [NEL-(K2)-2019(02)] was deposited at the Herbarium of School of Biological Sciences, Universiti Sains Malaysia.

### Preparation of Kratom Decoction

The collected fresh kratom leaves (1 kg) were washed with tap water and ripped into small pieces before placing them into a boiling pot of water (4 L). The leaves were brewed for approximately 2 h at constant heat until the volume was reduced to approximately one-third of the initial volume. After that, the solution (1 L) was left to cool, filtered, and freeze-dried to yield a lyophilized kratom decoction extract. The lyophilized extract was kept at −80°C before high performance liquid chromatography (HPLC) analysis and toxicity evaluation.

### Extraction and Isolation of Mitragynine and Speciociliatine

Mitragynine (1) and speciociliatine (2) were extracted and purified from the fresh *M. speciosa* leaves according to the method described in our previous study (Chear et al., 2021). The detailed isolation procedures and spectroscopic data are provided in Supplementary Methods and Supplementary Figures S1–6.

### HPLC Analysis

#### Chemicals and Reagents

The reference standards: mitragynine (1) and speciociliatine (2) (purity ≥97%) (Supplementary Figures S7, S8) were extracted according to the method described by Saref et al. (2019). Solvents—acetonitrile and methanol used for analysis were of LC grade (Merck, Germany). Formic acid (98–100%) was purchased from Merck (Germany). Deionized water (18.2 MΩ) was used for the HPLC analysis.

### Analytical Method

The content of mitragynine (1) and speciociliatine (2) in the prepared kratom decoction sample were determined using a validated HPLC method as described in our previous study (Saref et al., 2019). The detailed HPLC analytical methodology is provided in Supplementary Methods.

### Fish Embryo Acute Toxicity Test

The stock solutions of kratom decoction (2 mg/ml), mitragynine (200 μg/ml), speciociliatine (200 μg/ml), and morphine (200 μg/ml), were prepared in 0.1% dimethyl sulfoxide (DMSO). A series of working concentrations ranging from 1,000–62.5 μg/ml (kratom decoction) and 100 to 3.125 μg/ml (mitragynine, speciociliatine, and morphine) were prepared by serial dilution of the stock solution. System water was used as negative control and 0.1% DMSO as solvent control, whereas 20 μg/ml doxorubicin was used as a positive control. The FET test was performed according to the Organization for Economic Co-operation and Development (OECD) TG 236 guideline (OECD, 2013). Briefly, forty embryos (n = 40, <3 hpf) were pre-exposed to either solvent, negative or positive controls, or kratom decoction, mitragynine, speciociliatine, and morphine at various test concentrations in the petri dishes to optimize the exposure duration. Then, embryos were observed under the microscope (Olympus SZ61 Zoom Stereo Microscope), and fertilized embryos (*n* = 20) that reached the blastula stage with normal cleavage pattern were randomly transferred to 24-well plates, one embryo in each well with 1.5 ml of the test sample. Next, well plates were incubated at 26 ± 1°C under a 14 h light: 10 h dark cycle. The test samples were renewed on the daily basis (semi-static exposure). The tests were performed in triplicate. Lethality parameters, such as coagulation of embryos, lack of somite formation, non-detachment of the tail, and lack of heartbeat and sub-lethal parameters include pericardial oedema, yolk sac oedema, spinal curvature (kyphosis, lordosis or scoliosis), heartbeat and hatching rate at 24, 48, 72 and 96 hpf were examined under zoom stereo microscope. The heart rate of larvae (*n* = 5) was counted for 15 s using a stopwatch under the stereomicroscope when the larvae were immobile, and then multiply by 4 to obtain the beats per minute. The sub-lethal morphological effect was expressed as the percentage of embryos with malformation over total alive embryos at 24, 48, 72, and 96 hpf (Nagel, 2002; Blahova, 2020).

### Statistical Analysis

Statistical analysis was performed using the software Graph Pad Prism. 5. Data were expressed as mean ± standard deviation (SD). Concentration-response curves were used to determine the lethal concentration, LC_50_ value. The mortality rate at 24, 48, 72, and 96 hpf were analyzed using a two-way repeated-measure ANOVA, followed by Bonferroni *post hoc* test. The hatching rate, heartbeat, and sub-lethal morphological effect data were analyzed using one-way ANOVA followed by Dunnett’s *post hoc* test. Probability values of less than 5% (*p* < 0.05) are considered significant.

## Results

### Mitragynine and Speciociliatine Content

Based on HPLC analysis, the amount of mitragynine (1) and speciociliatine (2) detected in the prepared kratom decoction (lyophilized extract, 1,000 μg/ml) were 37.63 ± 3.01 and 5.49 ± 0.37 μg/ml, respectively. The HPLC chromatograms of lyophilized kratom extracts and its detected mitragynine 1) and speciociliatine 2) are provided in Supplementary Figure S9.

### Mortality

The test was validated according to OECD 236 guideline criteria, as shown by less than 5% mortality rate in system water (negative control) and 0.1% DMSO (solvent control), whereas 100% mortality rate in 20 μg/ml doxorubicin (positive control) at 96 hpf (Supplementary Figure S11).

**FIGURE 1:**
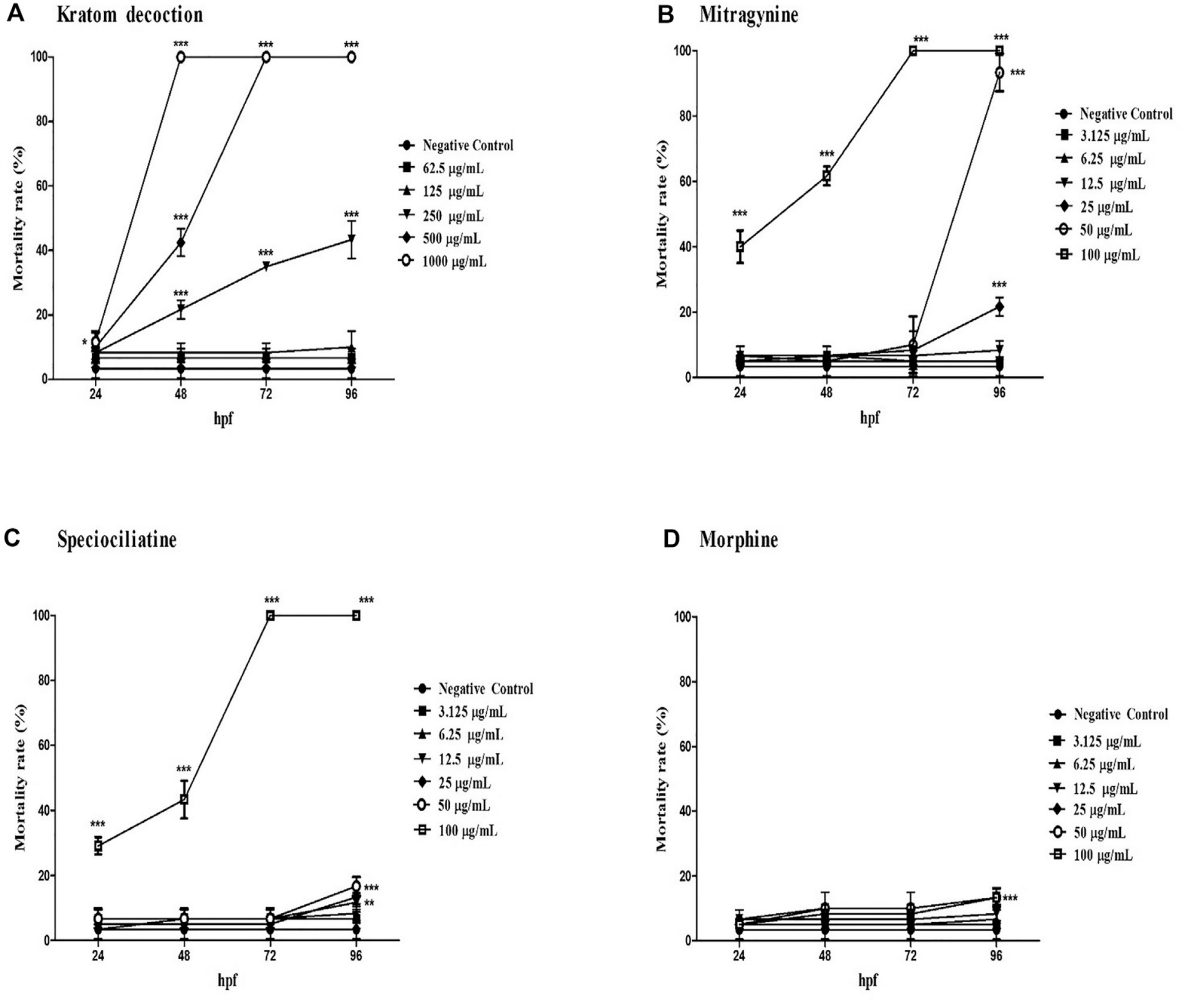
The mortality rate of zebrafish embryos exposed to **(A)** kratom decoction, **(B)** mitragynine, **(C)** speciociliatine, and **(D)** morphine at 24, 48, 72, and 96 hpf. Data are expressed as the mean ± SD. **p < 0.01, ***p < 0.001 significantly different from negative control group.

As shown in Figure 1, kratom decoction, mitragynine, and speciociliatine caused mortality of zebrafish embryos in a time and concentration-dependent manner. Zebrafish embryos exposed to 1,000 μg/ml kratom decoction showed 100% mortality at 48 hpf, similar finding for 500 μg/ml at 72 hpf. At 250 μg/ml, the mortality rate was gradually increased from 48 hpf to 96 hpf (*p* < 0.001, versus negative control group). With regards to alkaloid compounds, both mitragynine and speciociliatine at the highest concentration (100 μg/ml) killed 100% of the embryos at 72 hpf. Besides that, the mortality rate of embryos in both mitragynine and speciociliatine groups at concentrations 50 and 25 μg/ml was significantly increased at 96 hpf (*p* < 0.01, versus negative control group). Mitragynine at concentrations of 50 and 25 μg/ml killed 93.33% and 21.67% of the embryos, respectively, whereas the mortality rate of speciociliatine at 50 and 25 μg/ml were 16.67% and 13.33%, respectively. These results indicate that speciociliatine is safer than mitragynine. In morphine-exposed embryos, the highest concentration (100 μg/ml) significantly increased the mortality rate at 96 hpf in comparison to the negative control group (*p* < 0.001).

The concentration-response curve of kratom decoction, mitragynine, speciociliatine, and morphine for mortality rate at 96 hpf are shown in Supplementary Figure S12. The LC_50_ of kratom decoction and mitragynine at 96 hpf were 260.68 μg/ml and 32.01 μg/ml, respectively. We were unable to calculate the actual LC_50_ value of speciociliatine since it showed an exponential increase of mortality rate at 100 μg/ml concentration, hence the estimated LC_50_ of speciociliatine at 96 hpf from the concentration-response curve was 79.86 μg/ml. For morphine, the highest mortality rate recorded was less than 20%, and it is not possible to calculate the LC_50_ value.

### Hatching Rate

**FIGURE 2:**
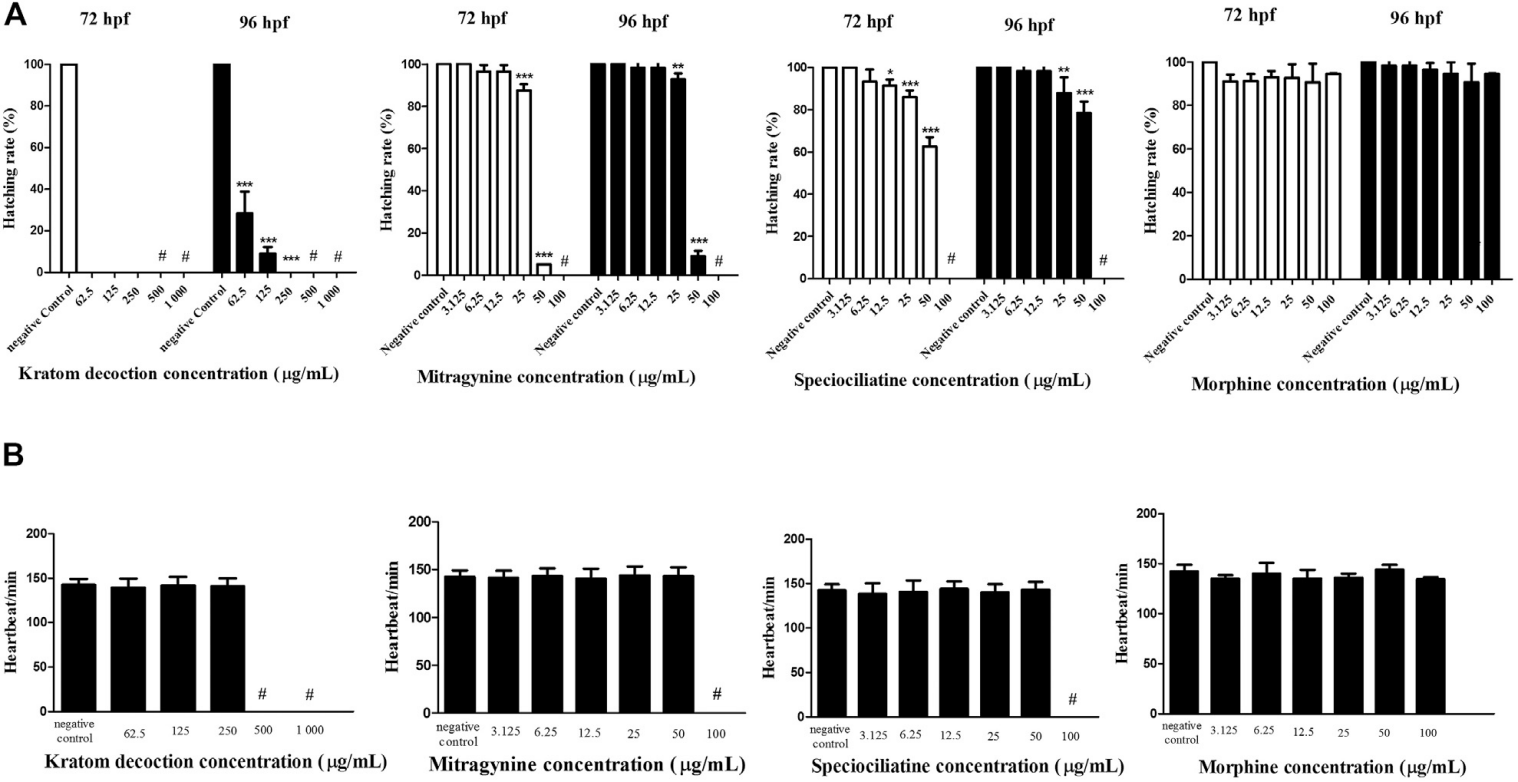
Zebrafish embryos exposed to kratom decoction, mitragynine, speciociliatine, and morphine. **(A)** hatching rate at 72 and 96 hpf and **(B)** heart rate per minute. Data are expressed as the mean ± SD *p < 0.05, **p < 0.01, ***p < 0.001 significantly different from the negative control group. ^#^ indicate not determined.

In general, zebrafish embryos started to hatch from 48–72 hpf (Kimmel et al., 1995). As shown in Figure 2, untreated embryos (negative control group) showed a 100% of hatching rate at 72 hpf. One-way ANOVA revealed that kratom decoction at 250, 125 and 62.5 μg/ml affect the hatching rate of zebrafish embryos, as shown by 0% hatching rate at 72 hpf (Figure 2A). At 96 hpf, the highest concentration of kratom decoction (250 μg/ml) resulted in a 0% hatching rate, indicating complete inhibition of hatching (*p* < 0.001, versus negative control group). In addition, the percentage of embryos hatched was only 9.06% and 28.36% in the group treated with 125 and 62.5 μg/ml of kratom decoction at 96 hpf, respectively (*p* < 0.001). These data indicated concentration-dependent delayed hatching in the kratom decoction-treated groups in comparison to the control group. Hatching inhibition was also found in higher concentrations of mitragynine and speciociliatine (50 and 25 μg/ml) treated groups at 72 and 96 hpf, respectively (*p* < 0.01, versus negative control group). Morphine did not affect the hatching rate of zebrafish embryos at 72 and 96 hpf.

### Heart Rate

The heart rate per minute of zebrafish embryos at 96 hpf is shown in Figure 2B. One-way ANOVA showed that exposure of kratom decoction, mitragynine, speciociliatine, and morphine until 96 h did not affect the heart rate of exposed zebrafish embryos, suggesting that kratom or morphine did not hindered the development of cardiovascular system in zebrafish embryos (*p* > 0.05). The heart rate of kratom decoction (1,000 and 500 μg/ml), mitragynine, and speciociliatine (100 μg/ml) was not determined, as there were no surviving embryos at 96 hpf.

### Morphological Malformations

**FIGURE 3:**
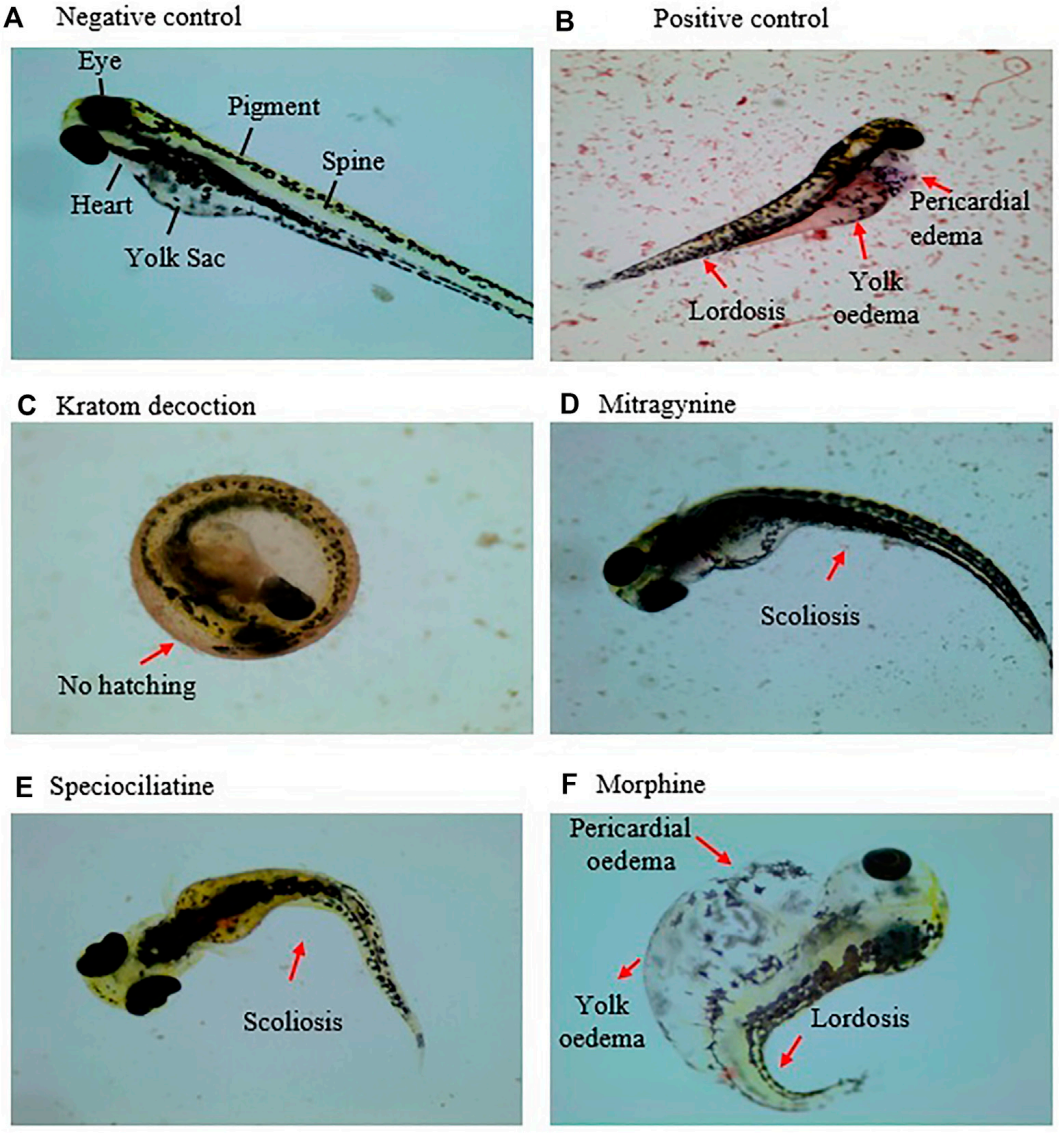
Morphology of the zebrafish embryo at 96 hpf exposed to **(A)** system water (normal morphology), **(B)** doxorubicin, 20 μg/ml, **(C)** kratom decoction, **(D)** mitragynine, **(E)** speciociliatine, and **(F)** morphine. Morphological malformations are denoted with red arrows.

Table 1; Figure 3 show the sub-lethal morphological alterations in zebrafish embryos exposed to kratom decoction, mitragynine, speciociliatine, and morphine for 96 h, respectively. Zebrafish embryos exposed to system water or 0.1% DMSO showed normal morphology features with normal body shape, straight spine, pigmented body, and round yolk sac (Figure 3A). Meanwhile, embryos exposed to 20 μg/ml doxorubicin displayed pericardial oedema, spinal curvature (lordosis), and small eyes (Figure 3B). Kratom decoction at concentrations ≤250 μg/ml did not show any morphological malformations, except for hatching inhibition compared to the negative control group (*p* > 0.05, Figure 3C). As shown in Figure 3D, mitragynine at 50 μg/ml caused spinal curvature (scoliosis) in zebrafish embryos (*p* < 0.01, versus negative control group). Meanwhile, the embryos exposed to speciociliatine at the concentration ≥25 μg/ml exhibited signs of scoliosis (*p* < 0.01, versus negative control group, Figure 3E). Morphine at the concentration ≥6.25 μg/ml showed pericardial oedema, meanwhile, 100 μg/ml exhibited spinal curvature (lordosis) and yolk oedema as compared to the negative control group (*p* < 0.05, versus negative control group, Figure 3F).

**TABLE 1:** Morphological malformations in zebrafish embryos at 96 hpf.

**Concentration (µg/ml)**	**Morphological** **abnormalities (%)**		
	**Pericardial oedema**	**Yolk oedema**	**Spinal curvature**
Negative Control	0	0	0
Solvent Control	0	0	0
Positive control	100 ± 0.00	100 ± 0.00	67.18 ± 20.59
**Kratom decoction**			
31.25	0	0	0
62.5	0	0	0
125	0	0	0
250	0	0	0
500	^a^	^a^	^a^
1000	^a^	^a^	^a^
**Mitragynine**			
3.125	0	0	0
6.25	0	0	0
12.5	0	0	0
25	0	0	3.92 ± 3.40
50	0	0	7.34 ± 2.80^**^
100	^a^	^a^	^a^
**Speciociliatine**			
3.125	0	0	0
6.25	0	0	0
12.5	0	0	0
25	0	0	12.48 ± 2.88^***^
50	0	0	25.05 ± 3.54^***^
100	^a^	^a^	^a^
**Morphine**			
3.125	5.36 ± 0.17	1.75 ± 3.04	5.26 ± 5.26
6.25	7.02 ± 3.04^*^	3.51 ± 3.04	5.46 ± 5.56
12.5	7.21 ± 3.38^*^	3.61 ± 3.13	5.36 ± 0.17
25	7.42 ± 3.21^*^	3.81 ± 3.31	7.42 ± 3.21
50	9.26 ± 3.21^**^	5.56 ± 1.09	7.41 ± 3.21
100	9.06 ± 3.05^**^	7.21 ± 2.87^*^	9.16 ± 3.38^*^

Data are expressed as the mean ± SD. *p < 0.05, **p < 0.01, ***p < 0.001 significantly different from the negative control group.

a indicate not determined.

## Discussion

With kratom having many medicinal applicability, it is important to ensure that the plant and its alkaloids are safe for human consumption. To date, there is no pre-clinical toxicity data on kratom decoction, despite it being used pervasively in the community. So far, only one study have managed to previously determine kratom extract effects in zebrafish embryos (Ramli et al., 2020). To the best of our knowledge, this study is among the first to investigate the embryotoxicity of kratom alkaloids both mitragynine and speciociliatine in comparison to morphine in zebrafish embryos.

This study demonstrates that acute embryonic exposure to kratom decoction, mitragynine, and speciociliatine affected survival, hatching, and body morphology of zebrafish embryos in a concentration and time-dependent manner, indicating that higher extract/compound concentrations and longer exposure times affect the zebrafish embryo development. Herein, kratom decoction at a concentration of ≥500 μg/ml caused 100% mortality of zebrafish embryos at 96 hpf. A previously published acute toxicity study reported that methanolic extract of kratom up to 1,000 mg/kg did not cause any mortality in mice (Harizal et al., 2010). This discrepancy is probably due to the high sensitivity of zebrafish embryos in their early development stages to external stimuli/exposure (Hill et al., 2005). Thus, there is a possibility that a high concentration of kratom decoction may be toxic to zebrafish embryos, but not to other species, and the toxicity level of the extract might also rely on the development stage of animals.

With regards to alkaloids, the LC_50_ value of mitragynine was 32.01 μg/ml at 96 hpf, while the estimated LC_50_ value of speciociliatine was 79.86 μg/ml, indicating that speciociliatine is relatively safer than mitragynine. However, kratom decoction showed lower embryotoxicity compared to its major active alkaloids with an LC_50_ value of 260.68 μg/ml. HPLC analysis reveals that the concentration of mitragynine and speciociliatine detected in the lyophilized kratom decoction was relatively low which was approximately 3.76 and 0.55% w/w of lyophilized powder, respectively. This suggests that the embryotoxicity observed in kratom decoction treatment might not be link to mitragynine and speciociliatine per se since their respective LC_50_ values (as a single agent) are far higher compared to kratom decoction (as a mixture). The embryotoxicity observed in kratom decoction might be due to the presence of other phytochemicals such as other indole and oxindole alkaloids, terpenes, flavonoids, phenolics, plant peptides, polysaccharides, etc. The single or synergic effect of these compounds in the overall embryotoxicity of kratom decoction cannot be ruled out as well, therefore this warrants further investigation. For morphine, we could not determine the LC_50_ value because the highest mortality rate recorded was less than 20%, indicating that morphine was relatively safe even at a higher concentration range up to 100 μg/ml. Altogether, kratom decoction, mitragynine, and speciociliatine at the highest dose display more toxic effects in terms of embryo survival, when compared to morphine.

Hatching is the most important process in the development stage of zebrafish and its retardation following exposure to chemical/drugs like environmental pollutants, nanomaterials, or natural product is a sign of sub-lethal toxicological effects on zebrafish embryos (Liu et al., 2014; De la Paz et al., 2017). In the present study, we found that exposure to kratom decoction significantly reduced the hatching rate of the zebrafish embryos. Notably, mitragynine and speciociliatine at concentrations of 25 and 50 μg/ml appeared to be associated with delayed hatching process. However, morphine did not affect the hatching of zebrafish embryos. In zebrafish, hatching enzyme 1 (HE1) is secreted from hatching gland cells (HGCs) to digest the outer chorion layer for the natural hatching process to occur (Zhou et al., 2009). The delayed hatching process observed in this study might be due to delayed HE1 secretion. A study by De la Paz et al. (2017) showed that triazole, fungicides inhibit the hatching process via reduction of HGCs secretion and administration of dopamine type 2 (D2) receptors antagonist able to reverse the effect of triazole. Their results suggest that the dopaminergic system regulates the secretion of HGCs in zebrafish embryos. Since mitragynine has been revealed to bind to the D2 receptor (Boyer et al., 2008), it is possible that kratom indirectly affects the HGCs secretion by regulating the dopaminergic system via D2 receptor-mediated signaling pathways. This notion warrants further investigation.

Morphological malformations such as spinal curvature, yolk oedema, and pericardial oedema are important sub-lethal parameters observed in zebrafish embryos when exposed to toxic chemicals (Chahardehi et al., 2020). An example, drugs such as alcohol and nicotine that are known to affect human fetal development have been reported to induce morphological defects in the zebrafish embryos (Lantz-McPeak et al., 2015). Spinal curvature can be further specified into three types: lordosis (spine curved inward), kyphosis (spine curved outward), and scoliosis (spine curved sideways) (von Hellfeld et al., 2020). In this study, spinal curvature (scoliosis) was observed in mitragynine (50 μg/ml) and speciociliatine (25 and 50 μg/ml) exposed groups. It is possible that mitragynine or speciociliatine may trigger neuroinflammation pathways in cerebrospinal fluid by activating pro-inflammation signals that in turn could lead to spinal deformities (Van Gennip et al., 2018). On the other hand, zebrafish embryos exposed to morphine had morphological malformations, including yolk oedema, pericardial oedema, and spinal curvature (lordosis) in a concentration-dependent manner. Correspondingly, Cadena et al. (2021) also observed the morphological malformations (yolk oedema, spine deformation, and tail deformation) in zebrafish embryos following exposure to 10 μg/ml morphine. The result indicates that morphine is more prone to cause abnormal embryonic development than kratom and its alkaloids mitragynine and speciociliatine in zebrafish. Taken together, it is plausible that the differences seen in the types of vertebral changes (i.e. demineralization, increased density, and alteration in intervertebral spacing) induced by kratom alkaloids and morphine could lead to different spinal curvature morphology (Eissa et al., 2009; Yashwanth et al., 2016). Overall, it is also apparent that kratom and morphine may act on a different pathway to induce toxicity during embryogenesis in zebrafish. However, the exact mechanisms involved is yet to be elucidated.

Overall, we have demonstrated that kratom (≥500 μg/ml) and its alkaloids mitragynine and speciociliatine (≥50 μg/ml) have certain undesirable effects on embryonic development by affecting survival, hatching, and body morphology of zebrafish embryos. This finding suggests that the potential risk of kratom intake during pregnancy on the development of the fetus is based on the fact that the early embryo developmental process of zebrafish is similar to humans. However, this notion should be interpreted with caution, and warrants further investigation in other animal models such as rodent.

## Data Availability

The raw data supporting the conclusions of this article will be made available by the authors, without undue reservation, to any qualified researcher.
