# Supplementary material for: Comparative Toxicity Assessment of Kratom Decoction, Mitragynine and Speciociliatine Versus Morphine on Zebrafish (Danio rerio) Embryos
Source: Front Pharmacol. 2021 Aug 20;12:714918. doi: 10.3389/fphar.2021.714918 (PMC8417521; doi:10.3389/fphar.2021.714918)
Supplement: Supplementary file 1 [file DataSheet1.docx]

Supplementary Material

**Methods**

**Extraction and isolation of mitragynine and speciociliatine**

Approximately 1.5 kg of fresh kratom leaves were dried at room temperature and ground into a powdered form using a grinding mill machine. The resulting 550 g of dried leaf powder was extracted with hot methanol (5 L) in a circulating water bath (45 °C) for 36 h according to the extraction procedure described by Chear et al. (2021). After the extraction process, the filtered solution was evaporated under reduced pressure to yield the crude methanol extract. The concentrated extract was then partitioned between hexane (3 x 2 L) and 10% acetic acid (4 L). The remaining aqueous-soluble portion was adjusted to pH 9.0 with 25% ammonia solution and extracted with chloroform (3 x 2 L). The chloroform-soluble portion was evaporated under reduced pressure to yield the alkaloid-enriched extract (5.6 g). The alkaloid extract was fractionated on a silica gel column chromatography (CC) (hexane-ethyl acetate – methanol, 100:0:0; 0:0:100, v/v) to give seven major fractions (F1 – F7). Mitragynine-rich fraction (F2) (2g) was further purified by a silica gel CC (hexane-ethyl acetate, 80:20, v/v) to yield pure mitragynine (**1**) (1.68 g). Speciociliatine (**2**) (60 mg) was purified from F6 by multiple silica gel CC (ethyl acetate – methanol, 100:0 to 0:100, v/v). The chemical identity of mitragynine (**1**) and speciociliatine (**2**) were examined by ^1^H & ^13^C NMR and GC-MS (**Supplementary Figure 1-6**). The purity level of these compounds was accessed by HPLC-PDA according to a validated method described by Saref et al. (2019).

**Analytical method**

The content of mitragynine (**1**) and speciociliatine (**2**) in the prepared kratom decoction sample were determined using a validated HPLC method as described in our previous study (Saref et al., 2019). In brief, the HPLC analysis was performed on an Agilent 1200 series HPLC system coupled to a photodiode array detector (Agilent, CA, USA). A stock solution of lyophilized extract (1000 μg/mL) was prepared in methanol and centrifuged to remove debris or undissolved particles. The compound separation was achieved on an Inertsil C8-3 column (4.6 x 150 mm, 5 μm) (GL Sciences Inc., Japan). The mobile phase was a mixture of 0.1% formic acid (**A**) and acetonitrile (**B**) running at a gradient method with a flow rate of 1 mL/min. The programmed gradient method is summarized in Table 1. The total run time was 27 min, and the sample injection volume was 10 μL. Mitragynine (**1**) and speciociliatine (**2**) were detected using an Agilent photodiode array detector at λ_max_ 250 nm. The targeted compounds (**1**) and (**2**) were identified by comparing their HPLC retention times and UV profiles with those of reference standards, respectively. Data analysis was conducted using the ChemStation LC3D software.

**Table 1:** Programmed HPLC gradient method

| **Time** | **0.1% Formic acid (A)** | **Acetonitrile (B)** |
| --- | --- | --- |
| **2** | 80 | 20 |
| **7** | 70 | 30 |
| **12** | 50 | 50 |
| **17** | 0 | 100 |
| **20** | 80 | 20 |
| **27** | 80 | 20 |

**Preparation of calibration curves**

The stock solution of mitragynine (**1**) and speciociliatine (**2**) (1000 μg/mL) was prepared in methanol, respectively. A mixture of mitragynine and speciociliatine stock solution (200 μg/mL each) was then prepared and further diluted into a serial of working solution 100, 50, 25, 12.5, 6.25, 3.125, and 1.56 μg/mL, respectively. Method accuracy (% recovery) was evaluated by spiking different concentrations of mixed standards (50 μg/mL and 25 μg/mL) into a diluted kratom decoction sample (750 μg/mL). For precision, three different concentrations of mixed standards (high, medium, and low) were analysed in triplicates, and the result was expressed as relative standard deviation (% RSD).

**Results**

**HPLC profiling of the prepared kratom decoction**

A total of 1000 mL of concentrated kratom decoction (extracted from 1 kg fresh kratom leaves) was lyophilized to yield 3.62 g of powdered extract. An aliquot of 1000 μg/mL of decoction extract was analysed using a validated HPLC-DAD method as described by Saref et al. (2019). The HPLC chromatograms for kratom decoction (lyophilized extract) and the detected alkaloids - mitragynine (**1**) and speciociliatine (**2**) are given in **Supplementary Figure 9**. The chromatographic peak of mitragynine and speciociliatine was identified by matching their retention times with those of reference standards, respectively. The UV profile of mitragynine and speciociliatine standards and their corresponded peaks in the lyophilized extract is provided in **Supplementary Figure 10**. Based on HPLC analysis, mitragynine (**1**) and speciociliatine (**2**) were detected at retention time, 8.38 and 9.39 min, respectively.

**Calibration curve and method validation**

A validated HPLC-DAD method was employed for the quantification of mitragynine (**1**) and speciociliatine (**2**), the major opioid-like alkaloids in kratom decoction (Saref et al., 2019). A linear calibration curve of mixed mitragynine (**1**) and speciociliatine (**2**) standards was obtained between 1.56 to 100 μg/mL. The mean equation for mitragynine (**1**) and speciociliatine (**2**) was y = 11.052× + 9.7423 and y = 11.153× + 5.3711, respectively. Both (**1**) and (**2**) had excellent correlation coefficients, R^2^ ≥ 0.9998. The recovery rate (%) of mitragynine (**1**) and speciocilitine (**2**) ranged from 96.81 to 102.89%, respectively in all the spiked samples. The intra-day precision (% RSD) for both (**1**) and (**2**) was below 5% (1.36 - 4.96%).

**Characterization of mitragynine (1) and speciociliatine (2)**

Mitragynine (**1**) was isolated as a yellow amorphous powder. EI-MS spectrum (Supplementary Fig. 1) of (**1**) exhibited a molecular ion peak at *m/z* 398.3 with major fragment ions at 214 (100), 397 (76), 398 (66), 383 (44), 186 (29), 269 (28), 199 (28), 200 (27), 215 (15), 399 (15). The ratio of 397(398):383 (molecular ion: demethylated ion) of (**1**) was > 1, indicating that its C-3 position has an *S*-orientation (Wang et al., 2014). The ^1^H and ^13^C spectra (Supplementary Figs. 2 and 3) further revealed that the C-20 position of (**1**) has an *S*-orientation based on the following indications: (a) deshielded H-17 proton (δ_H_ 7.46, s); and (b) chemical shift of C-18 at δc 12.88 (Flores-Bocanegra et al., 2020). The spectroscopic data (^1^H, ^13^C & MS) of mitragynine was also compared with that reported in published literature (Flores-Bocanegra et al., 2020; Sharma et al., 2019; Wang et al., 2014).

Speciociliatine (**2**) was isolated as a light orange amorphous powder. EI-MS spectrum (Supplementary Fig. 4) of (**2**) had a molecular ion peak at m/z 398.3 with characteristic fragment ions at 214 (100), 383 (54), 397 (36), 398 (34), 268 (25), 186 (22), 199 (22), 200 (20), 215 (15), 384 (136). The mass and fragment ions of (**2**) was identical to mitragynine (**1**), suggesting that (**2**) is an isomer of mitragynine. However, the ratio of 398 (397): 383 was less than 1, indicating that the C-3 position of (**2**) is in the *R*-orientation (Wang et al., 2014). The orientation of C-3 position of (**2**) was then assigned to *S* orientation due to the characteristic proton and carbon signals at H-17 (δ_H_ 7.42, s), and C-18 (δc 12.63), respectively, which is similar to mitragynine (15*S*, 20*S*) (Supplementary Fig. 4) (Flores-Bocanegra et al., 2020). The spectroscopic data (^1^H, ^13^C & MS) of speciociliatine was also compared with that reported in published literature (Flores-Bocanegra et al., 2020; Obeng et al., 2020; Wang et al., 2014).

**Supplementary Figure 1**: EI-MS spectrum of mitragynine (**1**)


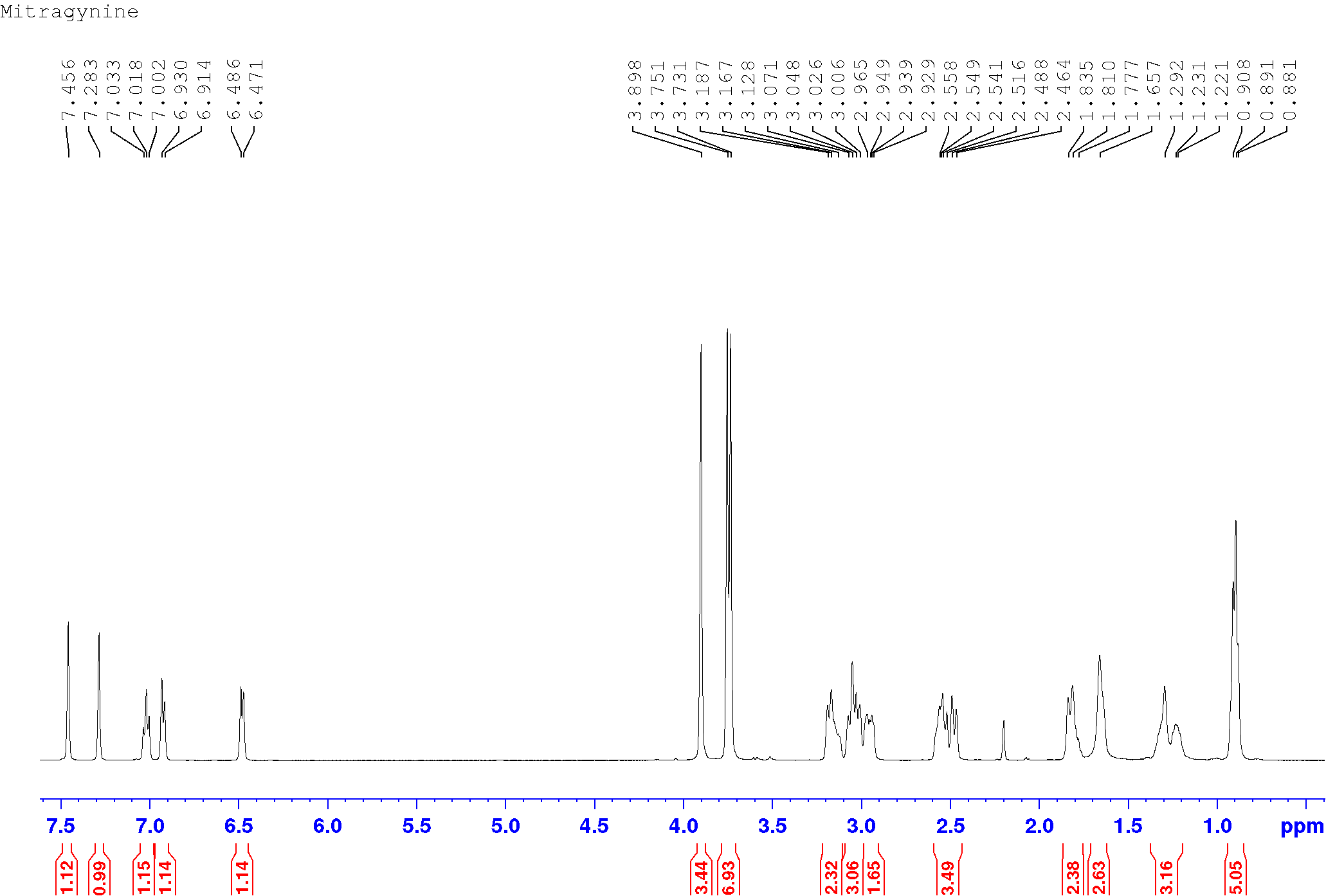


**Supplementary Figure 2**: ^1^H NMR spectrum of mitragynine (**1**) (CDCL_3_, 500 MHz).


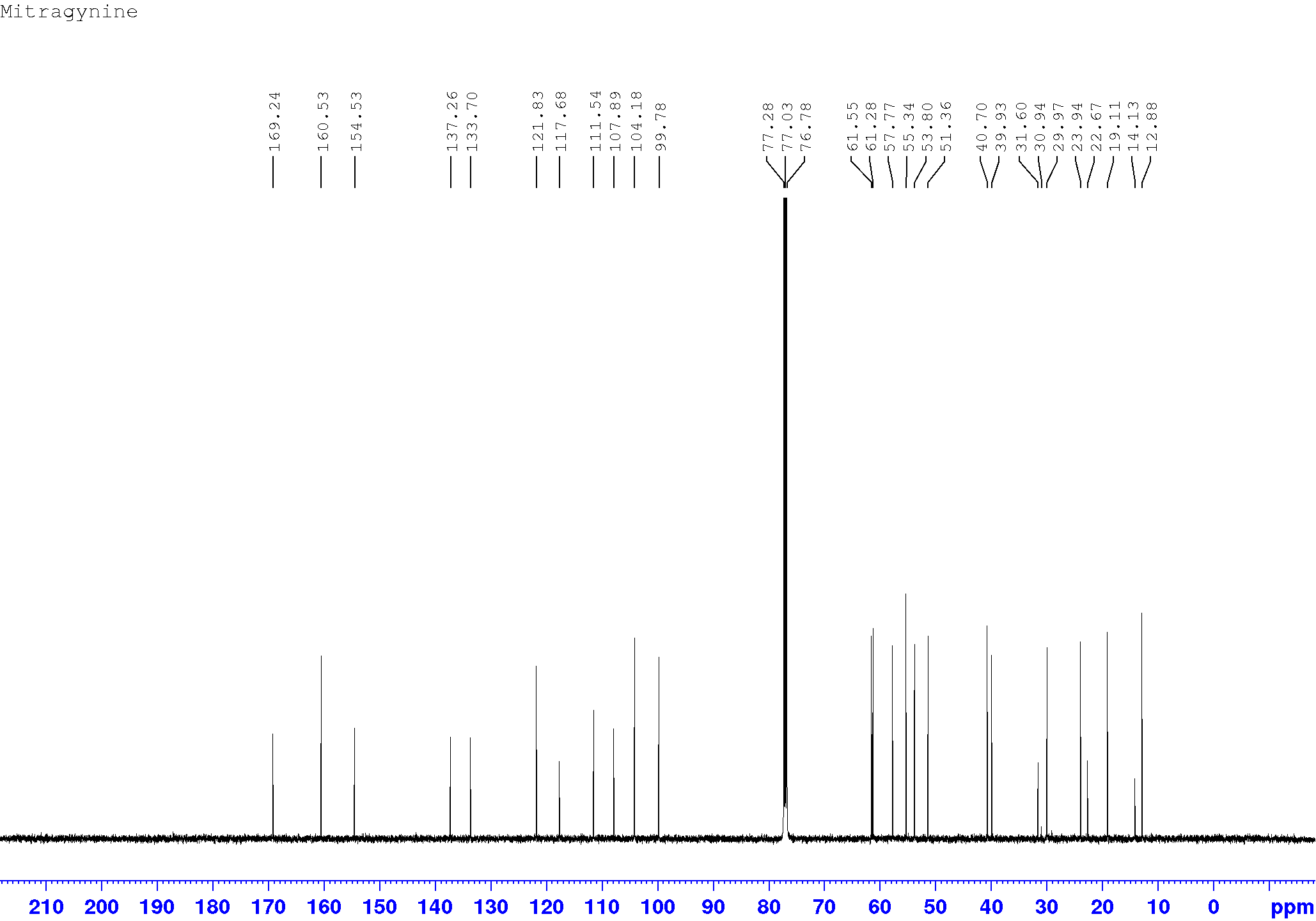


**Supplementary Figure 3**: ^13^C NMR spectrum of mitragynine (**1**) (CDCL_3_; 125 MHz)


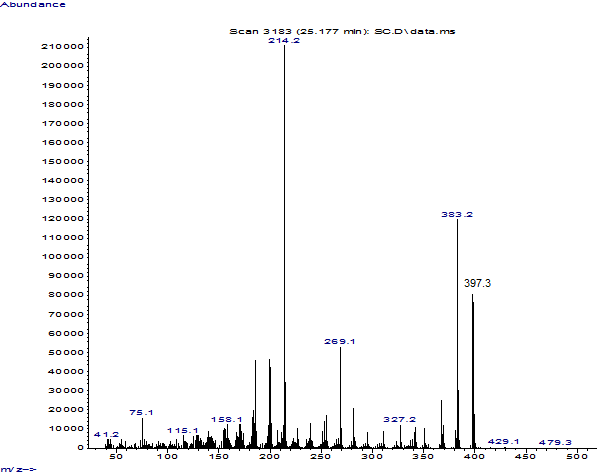


**Supplementary Figure 4**: EI-MS spectrum of speciociliatine (**2**)


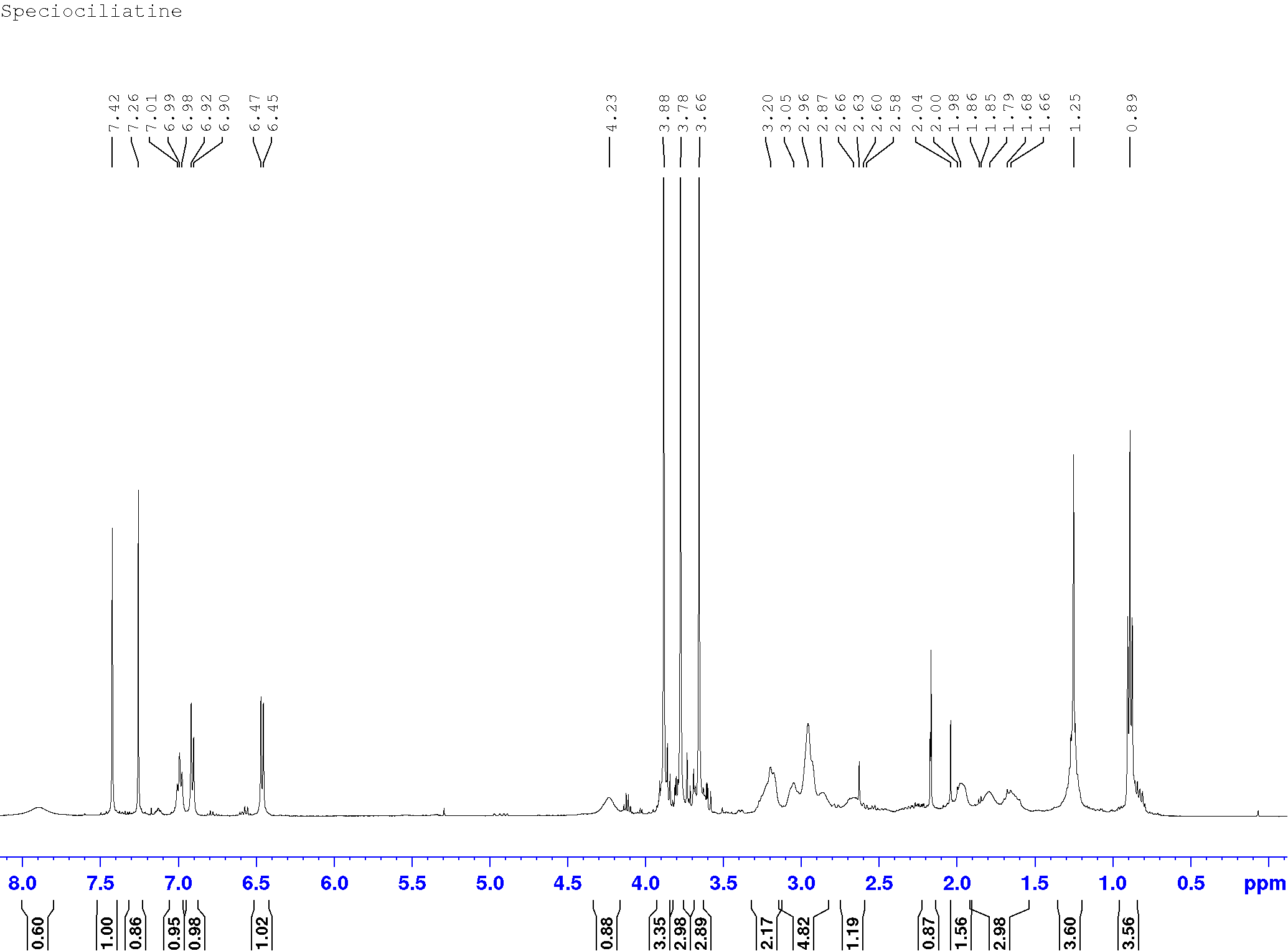


**Supplementary Figure 5**: ^1^H NMR spectrum of speciociliatine (**2**) (CDCL_3_, 500 MHz).


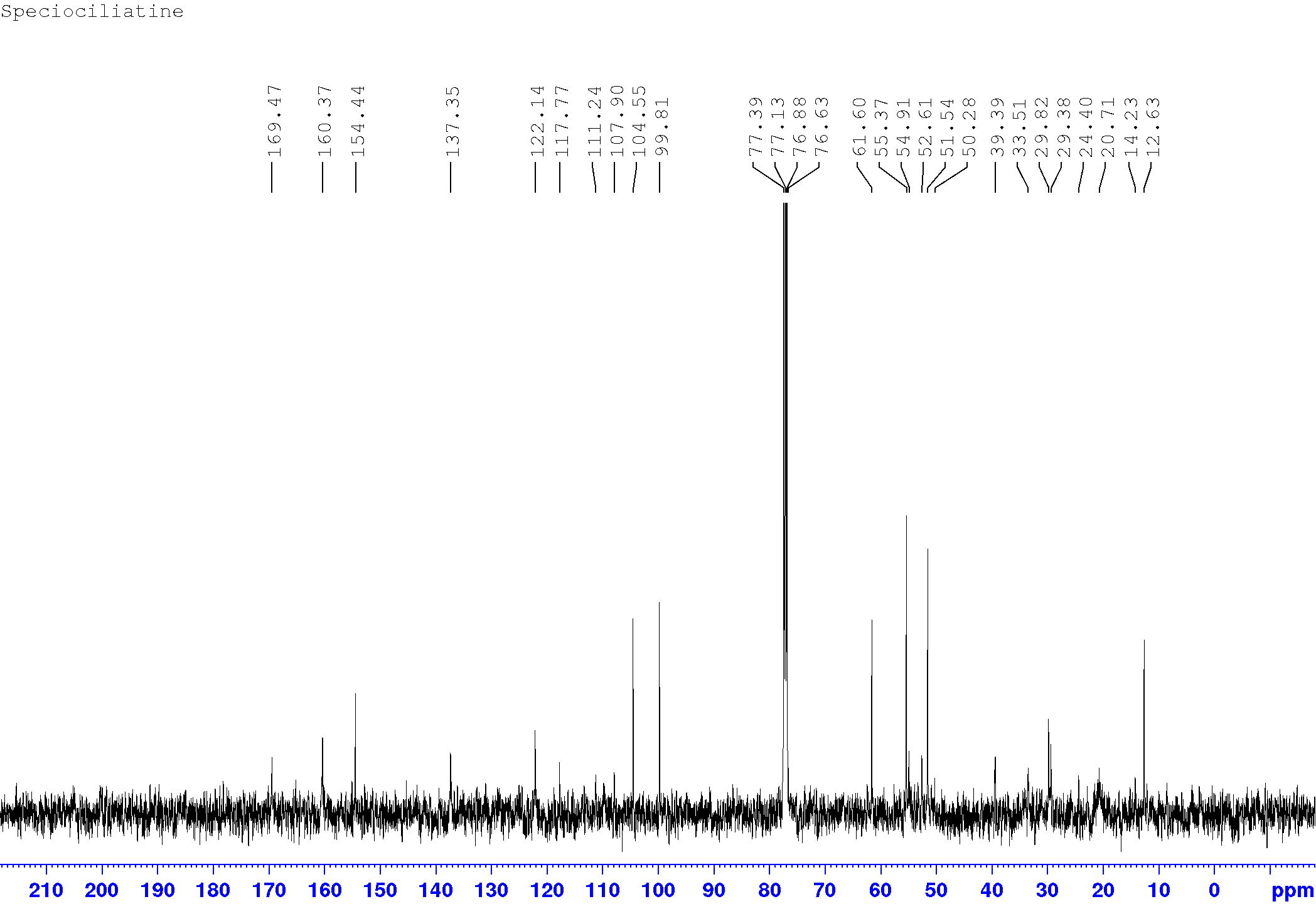


**Supplementary Figure 6**: ^13^C NMR spectrum of speciociliatine (**2**) (CDCL_3_; 125 MHz)

| # | Time | Area | Height | Width | Area% | Symmetry |
| --- | --- | --- | --- | --- | --- | --- |
| 1 | 7.63 | 28.8 | 2.2 | 0.1887 | 2.107 | 0.552 |
| 2 | 8.513 | 1337.2 | 165.5 | 0.1302 | 97.893 | 0.873 |

**Supplementary Figure 7**: HPLC chromatogram and purity of mitragynine (**1**)

| # | Time | Area | Height | Width | Area% | Symmetry |
| --- | --- | --- | --- | --- | --- | --- |
| 1 | 9.069 | 18.7 | 1.6 | 0.1635 | 1.834 | 1.762 |
| 2 | 9.386 | 1003.3 | 142.5 | 0.1121 | 98.166 | 0.897 |

**Supplementary Figure 8**: HPLC chromatogram and purity of speciociliatine (**2**)


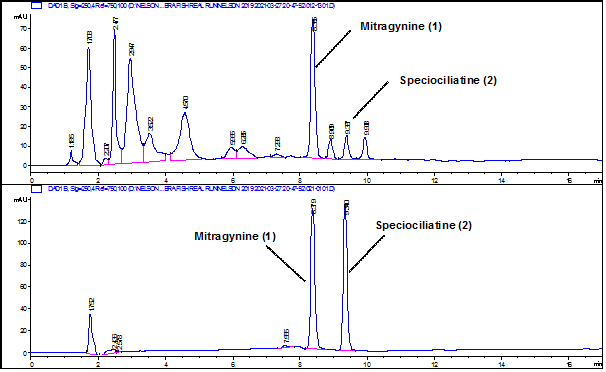


**Supplementary Figure 9:** A representative chromatogram of lyophilized kratom decoction (1000 µg/mL) and mixed standards - mitragynine (**1**) and speciociliatine (**2**) (100 µg/mL).


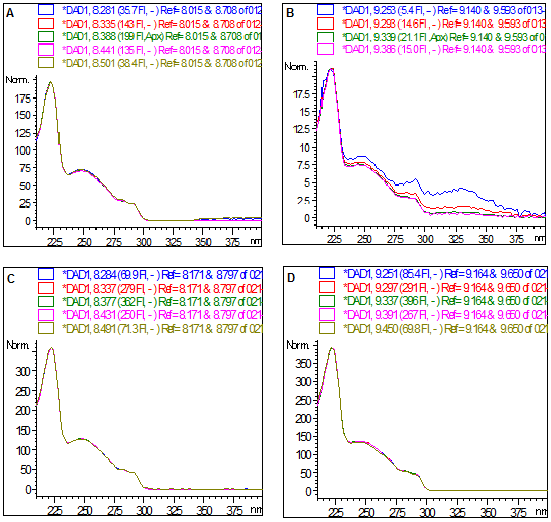


**Supplementary Figure 10:** UV profile of (**A**) mitragynine and (**B**) speciociliatine detected in lyophilized kratom decoction; (**C**) mitragynine and (**D**) speciociliatine standards.


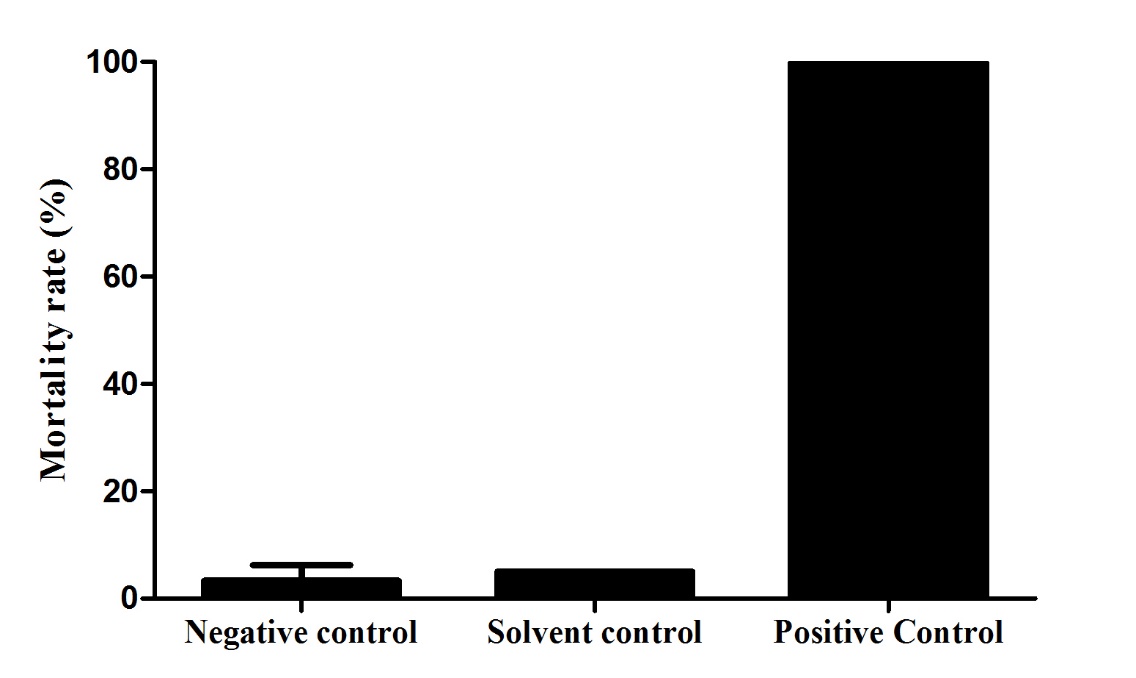


**Supplementary Figure 11:** The mortality rate of zebrafish embryos exposed to system water (negative control), 0.1% DMSO (solvent control), and 20 µg/mL Doxorubicin (positive control).


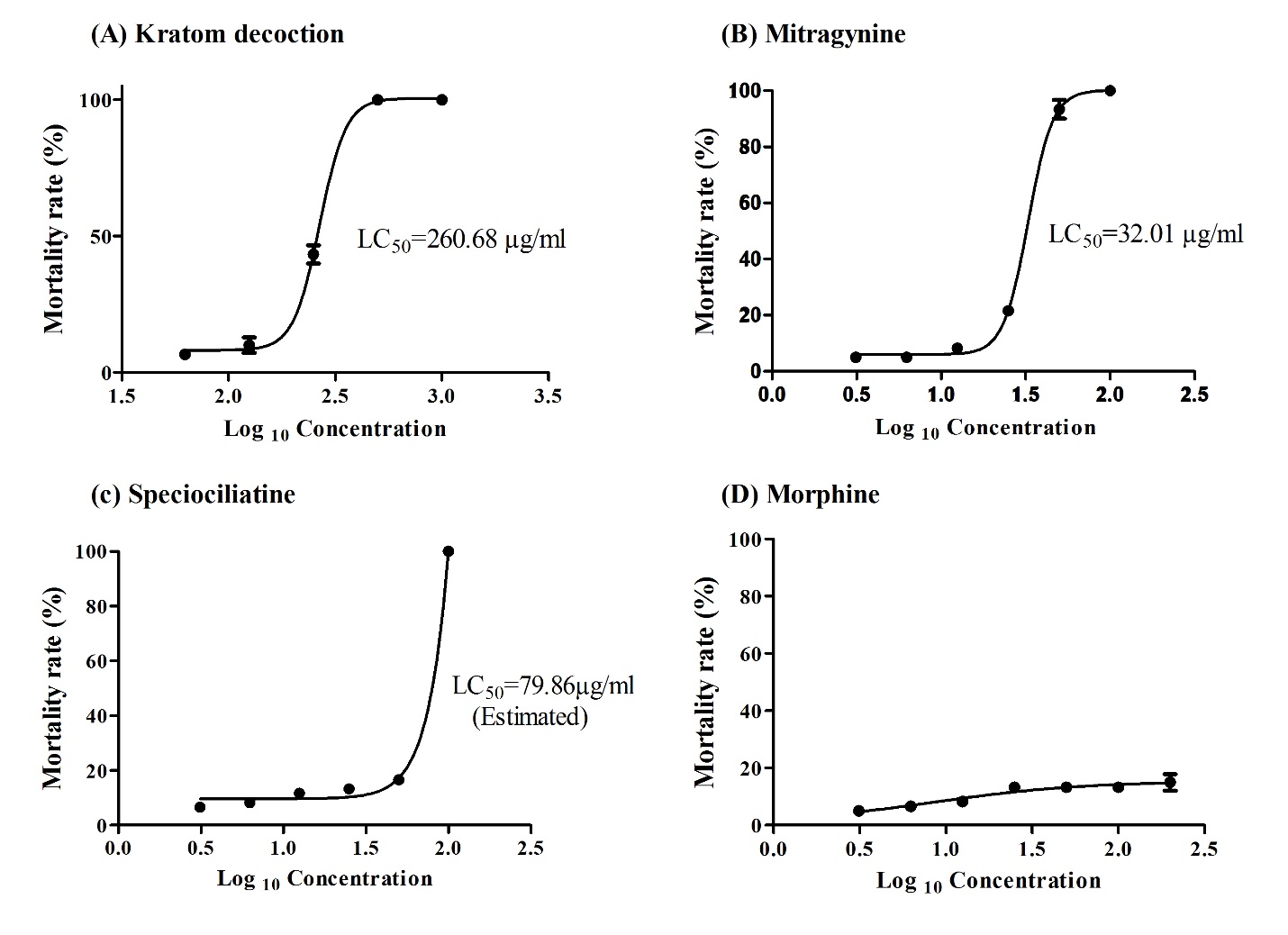


**Supplementary Figure 12:** The concentration-response curve of (A) kratom decoction, (B) mitragynine, (C) speciociliatine, and (D) morphine for mortality rate at 96 hpf.
